# Ant Distribution in Relation to Ground Water in North Florida Pine Flatwoods

**DOI:** 10.1673/031.012.11401

**Published:** 2012-10-07

**Authors:** Walter R. Tschinkel, Tyler Murdock, Joshua R. King, Christina Kwapich

**Affiliations:** ^1^ Department of Biological Science, Florida State University, Tallahassee, FL 32306-4370; ^2^ Biology Department, University of Central Florida, Orlando, FL 32816-2368

**Keywords:** ant abundance, ant ecology, community ecology, Formicidae, shading, soil moisture, soil profiles, vegetation

## Abstract

Longleaf pine savannas are one of the most threatened ecosystems in the world, yet are understudied. Ants are a functionally important and diverse group of insects in these ecosystems. It is largely unknown how local patterns of species diversity and composition are determined through the interaction of this dominant animal group with abiotic features of longleaf pine ecosystems. Here we describe how an important abiotic variable, depth to water table, relates to ant species distributions at local scales. Pitfall trapping studies across habitat gradients in the Florida coastal plains longleaf pine flatwoods showed that the ant community changed with mild differences in habitat. In this undulating landscape, elevation differences were less than 2 m, and the depth to the water table ranged from < 20 cm to 1.2 m. The plant species composing the ground cover were zoned in response to depth to water, and shading by canopy trees increased over deeper water tables. Of the 27 ant species that were analyzed, depending on the statistical test, seven or eight were significantly more abundant over a deep water table, eight to ten over a shallow one, and nine to eleven were not significantly patterned with respect to depth to water. Ant species preferring sites with shallow groundwater also preferred the shadier parts of the sites, while those preferring sites with deeper groundwater preferred the sunnier parts of the sites. This suggests that one group of species prefers hot-dry conditions, and the other cooler-moist. Factor analysis and abundance-weighted mean site characteristics generally confirmed these results. These results show that ant communities in this region respond to subtle differences in habitat, but whether these differences arise from founding preferences, survival, competition, or some combination of these is not known.

## Introduction

Longleaf pine-wiregrass savanna ecosystems of the southeastern United States of America contribute disproportionately to continental biodiversity, but are one of the most threatened ecosystems in the world. In spite of this, they are under-studied relative to other temperate ecosystems (Noss et al. 1995; Ford et al. 2008). Ants are conspicuous in these ecosystems, comprising a functionally important and diverse group of insects (Tschinkel 2002; Lubertazzi and Tschinkel 2003; King and Porter 2005). Because ants are of higher biomass in longleaf pine ecosystems compared to other ecosystem types in northern Florida, they are likely also one of the most important animal groups (King 2010; King unpublished data). Despite their importance in these longleaf pine ecosystems, it is largely unknown how they interact with abiotic features to determine local patterns of species diversity and composition.

There is the ample evidence that habitat characteristics are important in shaping ant communities. There are numerous examples of ant species associated with particular habitat features. Boomsma and De Vries (1980), Boomsma et al. (1987), and Galle (1991) found that ant species segregated strongly among particular habitats within successional dune ecosystems and on the Frisian Islands. Similarly, Dietrich and Wehner (2003) and Levings (1983) found that ant species distribution was affected by water availability, whereas du Merle et al. (1984) found that two sympatic *Lasius* species were separated by soil type. Soil type also plays a role in the distribution of *Pogonomyrmex* spp. (Johnson 2000). Wang et al. (2001) showed that the ant faunae of the Appalachian Mountains changed in relation to habitat characteristics, and Omelchenko and Zhigulskaya (1997) reported similar findings in Russia. Van Ingen et al. (2008) found abrupt changes in ant communities at the transition from forest to savannah. It is obvious that native ant communities are structured in large part by habitat. Every ant collector is of the persuasion that to find a given species of ant, one first goes to the habitat in which it is typically found. Books such as *Ants of North America* (2007) provide just such information to aid collectors. What is less obvious is how important different features, such as abiotic gradients of water availability, fire frequency, or soil characteristics, of particular ecosystems are in shaping the plant and animal communities. Furthermore, it is important to determine whether different taxa respond similarly or differently to the same gradients.

Longleaf pine-wiregrass ecosystems are firemaintained, and occur across a range of soil water availability determined by soil texture, topography, and depth to water table (Mitchell et al. 1999). Water availability is one of the primary factors governing plant community productivity (Ford et al. 2008), thus it is almost certainly a factor affecting animal populations. Other factors have already been shown to affect ant species in these ecosystems. For example, Tschinkel (1988) found two species of fire ant to be strongly segregated by the presence and absence of human-caused disturbance (forest roads), and Lubertazzi and Tschinkel (2003) found the ant community to vary along a wiregrass predominant gradient created by fire frequency. Seal and Tschinkel (2010) found that the populations of the fungus gardening ant, *Trachymyrmex septentrionalis*, in the Florida flatwoods contracted as a record drought ended.

The ground cover vegetation in subtly undulating pine flatwoods landscapes is strongly zoned in relation to depth to groundwater (which is rarely more than 1.5 m below the surface). It was observed that some ant species seemed to be zoned much like the ground cover plants, and occurred either predominately or only in the high spots over a deep water table, while other species occurred mostly over a shallow water table. It seemed reasonable that ground nesting ants, like plants (Ford et al. 2008), should respond to the water table, creating patterns of distribution. Thus, this observation was tested with transects that progressed from a wetland margin over the highest point of a flatwoods upland and down to the next wetland margin. The findings of this test are reported here.

## Materials and Methods

### Field sites

This research was carried out in the Apalachicola National Forest under US Forest Service Authorization ID APA663, issued 12-12-2002, amended 2/26/2004, expiring 2012. Most of the Apalachicola National Forest on the coastal plain south and west of Tallahassee, Florida consists of the pine flatwoods ecotype (Myers and Ewel 1990) developed on a gently undulating karst landscape, spodosol soils, and has a relief not usually exceeding 1.5 to 2 m. The plant species that typically compose the ground cover vegetation are listed in Appendix 1. Low areas are occupied by several types of wetlands, including titi, holly, gum, or cypress swamps, whereas the higher areas are dominated by longleaf pine forest with a ground cover of wiregrass, gallberry, runner oak, fetterbush, rusty staggerbush, shiny blueberry, and several less common woody shrubs (Appendix 1). Only the very highest points (1–2 m above the water table) are occupied by turkey oak, but such sites are relatively rare in the flatwoods. The ecotones between the uplands and wetlands support slash pine and a variety of grasses and insectivorous plants. About half of the Apalachicola National Forest is wetland.

The transition from wetland to upland includes conspicuous changes in plant cover, even though the elevation difference may total only a meter or two. The titi of the wetland margins gives way to dense fetterbush, then gallberry, shiny blueberry, and runner oak in various combinations, with an admixture of wiregrass and palmetto throughout. The ecotype is shaped by recurring, cool ground fires. When these fires are frequent, especially when they occur during the summer growing season, the ground cover is more dominated by wiregrass, and less dominated by shrubs. Reduced fire frequency and/or dormant season burns lead to a shrubbier ground cover dominated by runner oak, gallberry, shiny blueberry, or fetterbush, depending on proximity to wetlands.

The distribution of ant species in relation to depth to groundwater was tested by laying out three transects, each about 300 m long, that progressed from a wetland margin over the highest point of a flatwoods upland, and down to the next wetland margin. Three areas in which the highest points supported turkey oak were chosen, assuring that the full range of depth to water present in the flatwoods was sampled. These transects were designated by the US Forest Service management compartment in which they were located: 232 (transect centered at lat-long 30.324802, 84.520873), 233 N (centered at lat-long 30.329462, -84.530883), and 233 S (centered at lat-long 30.308122, -84.520544) (lat-long data from Google Earth).

### Determining the water table depth

A surveyor's level was used to map the elevations relative to the highest point along each transect. Sample plots were located at four roughly equal elevations along each transect so that each elevation was represented twice in each transect, once in one direction from the high point, and once in the other (that is, there were eight sample plots per transect). Thus, the wells were not spaced at equal linear distances along the transect. The two highest plots were at least 35 m apart. At each sample plot, a soil auger was used to bore a hole to below the water table, and a 10 cm diameter PVC pipe, fitted with a cap and screen bottom, was positioned in the hole and closed at ground level with a removable cap. The screen bottom allowed water, but not sand, into the pipe. The water level in these pipes represented the water table, and its depth from the surface was measured with a float and calibrated string. The water table was measured several times during 2005, and again from June to September 2009. The year 2005 was part of a multi-year drought, whereas 2009 was a more typical year.

**Figure 1.**
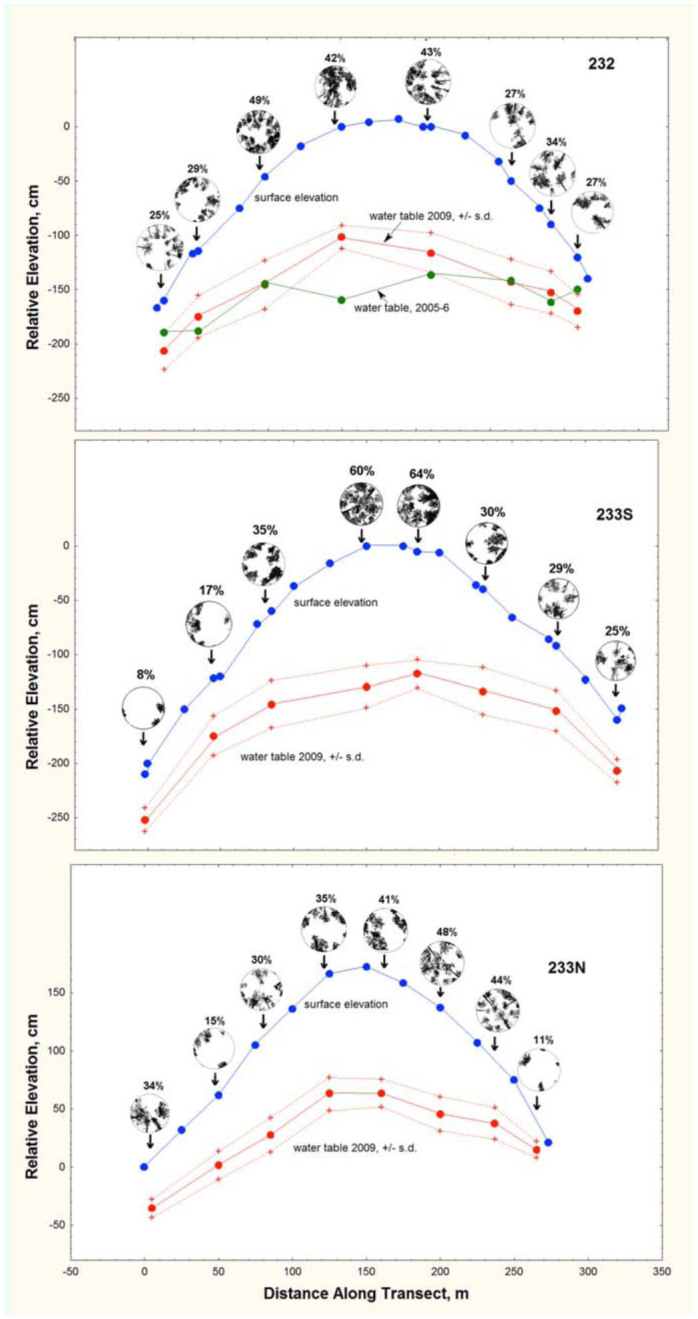
Profiles for the three transects, showing the surface elevation and the mean water table elevation in 2009, +/- s.d. The green line in the top panel shows the mean water table in 2005–6, a very dry year. The points on the water table curve show the locations of the test wells, as do the arrows. Depth to water increased with elevation, being maximum at the highest points. The water table fluctuated with rainfall, as shown by the standard deviation curves. Canopy images within the circles over each well location represent a view directly overhead, out to about 30°. Image was set to grey scale, and contrast was set to extreme. The percent canopy coverage given over each figure was calculated as the proportion of the total image pixels that were black. High quality figures are available online.

The well and plot locations are shown by arrows in Figure 1. Because the wells were located at four discrete depth classes, they were categorized as deep, upper medium, lower medium, and shallow. The mean and range of depth to water for these categories in 2009 was as follows: deep, mean = 108 cm, range 101–130 cm; upper medium, mean = 92 cm, range 86–100 cm; lower medium, mean = 59, range 53–62; shallow, mean = 45 cm, range 27–49 cm. None of the means overlapped between categories.

### Soil profiles

As the soil that was brought up while boring the wells changed color, a sample of it was packed onto a board, labeled with the depth range, and photographed. These images were used to generate the soil profile images for each well.

### Sampling the ant fauna

The ant fauna at each height above the water table was sampled with a 5 × 5 array of pitfall traps at 3 m spacing. The arrays (plots) were centered on the test wells, resulting in eight plots per transect. The pitfall traps were 85 mm long plastic vials with a 30 mm internal diameter, and were partially filled with ∼15 mm of non-toxic, propylene-glycol antifreeze (a preservative) sunk so that the upper lip was at ground level. An inverted, clear, plastic plate supported on three nails protected the traps from rain. The traps remained open for one week between the following dates: 232, September 3–15, 2008; 233 N, September 22–Oct. 3, 2008; 233 S, October 17–27, 2008.

After one week, traps were collected, capped, and returned to the laboratory. All ants were removed, preserved in alcohol, counted, and identified to species. A total of 600 traps captured 16,500 ants of 51 species, of which 27 were abundant enough for analysis. Appendix 2 lists the species captured, along with the total number of occurrences and specimens.

### Vegetation surveys

In June 2010, each sampled plot on each transect was surveyed for vegetation. The surveyor walked a series of parallel transects through each plot, stopping every two paces to identify the plant seen through a randomly oriented sighting tube (James and Shugart 1970). From the frequency of occurrence of each major ground-cover species, along with bare ground and litter, an estimate of percent coverage of each was calculated. These surveys took place two years after the ants were sampled, but in this exclusively perennial, woody vegetation, change is very slow. At most, minor differences in vegetation over this time span can be expected, most importantly, differences in the density and height of vegetation with elapsed time since a burn. Five canopy photographs were taken over each well, one directly upward, and the others angled about 30° toward each cardinal compass point. The composite images were used to assess the degree of shading from trees by converting each image to grey scale, setting the contrast to extreme and assessing the percentage of black pixels, which was expressed as percent canopy cover. For some analyses, the percent canopy shade was divided into four categories, from the least shady (1) to most shady (4).

### Data analysis

The basic data consisted of the number of ants of each species occurring in each trap, with the location of the trap being specified in relationship to the depth to the groundwater. Because the chief variable of interest was depth to groundwater (and, secondarily, local vegetation and canopy shade), the sum of each species within each plot was used in the analysis, but species yielding fewer than 15 individuals per transect were not used. Most analyses were thus carried out on a data set of 28 species. Raw abundances were analyzed by the non-parametric Kruskal-Wallace test, with depth category as the independent variable. The abundance of each species within each transect was ranked by plot, and both the abundances and the ranks were subjected to two-way ANOVA, with depth category and canopy category as the independent variables. The data were also subjected to a Mixed Procedure on SPSS, the outputs being residual maximum likelihoods. All these analyses are tabulated in Appendix 3. The three transects differed in both vegetation and overall ant fauna, and conversion to ranks for some analyses allowed all three transects to be analyzed together to reveal the patterns of occurrence in relation to depth to groundwater.

The raw abundances were also converted to the percent of the transect total of each species occurring in each plot. These percentages for all 27 species were subjected to factor analysis to extract groups of correlated species. Factor analysis was also applied to the ground cover vegetation data.

## Results

### Water table

The depth to the water table fluctuated with rainfall, was always greatest at the highest elevation of each transect, and was lowest adjacent to the wetlands. Figure 1 shows the mean water table for 2009. Depth to water can be seen as the difference between the elevation and water table curves. For transect 232, the mean water table for 2005–6, a very dry year, is also shown in Figure 1. Early June 2009 experienced unusually heavy rainfall, raising the water table for several weeks. During rainless periods, the water table sometimes fell as much as 2 cm per day. During long droughts, as in 2005, the water table was sometimes almost even with the elevation of the wetlands. The four depth-towater classes differed greatly in their mean depth, ranging from about 40 to 110 cm. In no category did the mean differ from the median by more than 3 cm. None of the extreme water levels overlapped between adjacent categories.

### Canopy shade and vegetation

**Figure 2.**
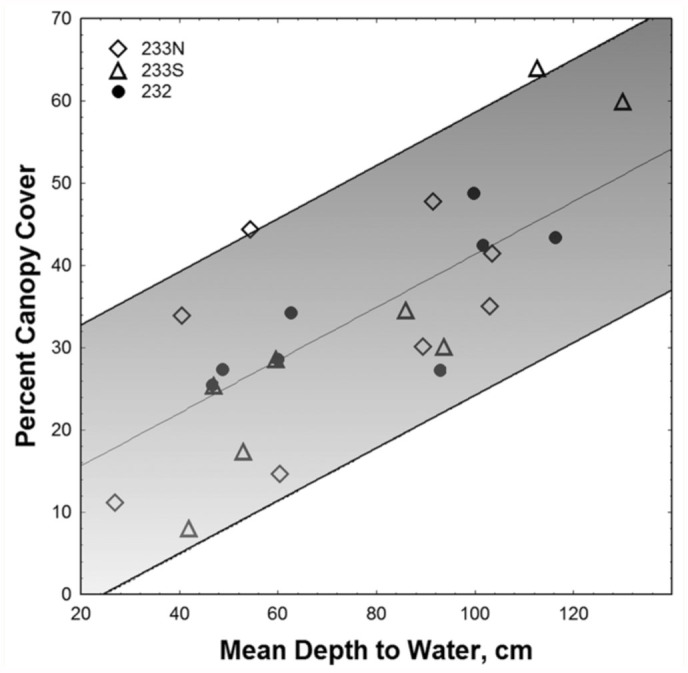
As elevation and depth to water increases, shading by canopy trees also increases. The shaded area between the two lines includes 96% of the regression data, i.e. two standard deviations around the mean regression; R^2^ = 0.56; F_1,22_ = 29.9 *p* < 0.00002. The symbols designate the Forest Service management compartment in which the transect was located. High quality figures are available online.

Figure 1 suggests that percent canopy shade increased with depth to water and elevation because turkey oaks were restricted primarily to the highest parts of the gradients, where they produced denser shade than the pines. Figure 2 shows that the relationship between shade and water table was strong, with percent canopy shade increasing about 4% for every 10 cm increase in depth to water, although there is considerable variation in shade for any particular depth to water (s.d.= 8%). Species composition of the ground cover also varied significantly with depth to water, as did soil composition. Both of these are presented in greater detail below.

### Ant distribution

**Figure 3.**
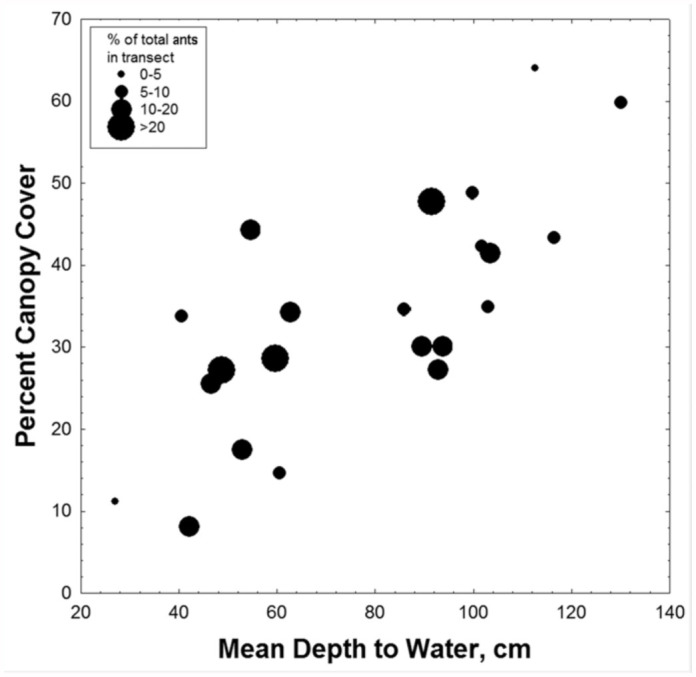
The distribution of all ants in relation to canopy shade and depth to water was not significantly patterned. Plot points are coded for the percent of all ants in the transect that occurred in each plot (2-way ANOVA, n.s.). Both extremes of the regression appear to harbor fewer ants, but these differences were not significant. High quality figures are available online.

In view of these variations in the distribution of vegetation, shade, depth to water, and soil characteristics along the flatwoods elevation gradients, parallel variation in soil-dwelling animals, such as ants, might be expected. Of the 52 species of ants that occurred in our samples, 27 species were represented by more than 15 individuals per transect, and were used in the analysis. Although the distribution of all species together was not related to depth to water or canopy shade (Figure 3; 2-way ANOVA: n.s.), preliminary analysis suggested that the distribution of several of these individual species, like that of plant species (see below), was strongly patterned in relation to the depth to water (and therefore to elevation), and, less so, percent canopy cover.

**Figure 4.**
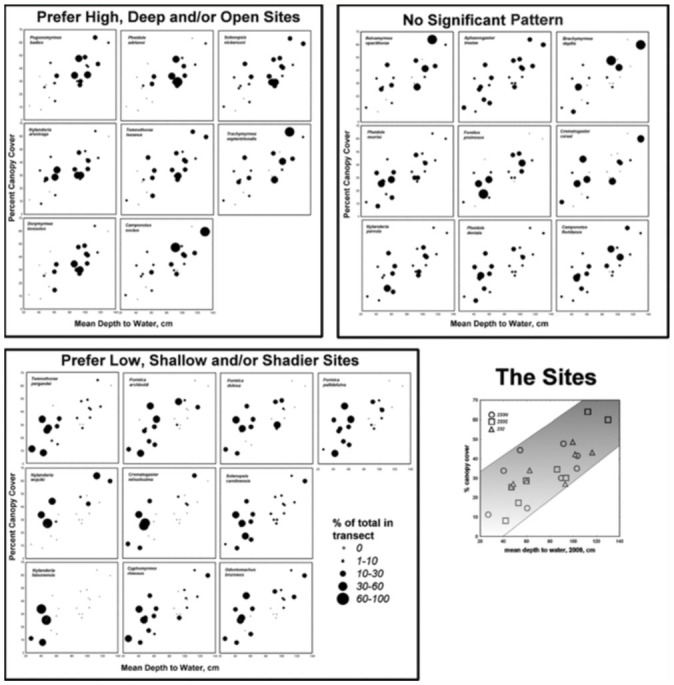
The distribution of species of ants in relation to depth to water and percent canopy cover. The points represent the depth and canopy of the test plots, coded for the percent of the transect total of each species that occurred in each plot. The concentration of large or small symbols in one region of these graphs indicates preference for particular depths to water and/or canopy shade. High quality figures are available online.

Further ANOVA (Type III sums of squares) and Mixed Procedure Analysis (SPSS) of both raw species abundances and abundance ranks revealed several species that were significantly patterned in relation to depth to water categories, shade categories, or both (Appendix 3). Appendix 3 also contains the results of a one-way non-parametric test (Kruskal-Wallis) by depth category alone, a test that combines the effects of depth to water and canopy shade. The table does not indicate the direction of the significant effects; instead, these distribution patterns can be seen in Figure 4, in which each test plot in the depth by canopy shade graph is coded for the percent of the total ants of each species in a transect that occurred in that plot. Some species showed consistent patterns in all analyses, while a few appeared marginal or unpatterned in some analyses but not others (Appendix 3; Figure 4 includes both consistent and marginal preferences). Such inconsistencies probably indicate weaker or more complex relationships, or differences in congruence of model assumptions and reality.

Figure 4 and Appendix 3 suggest a set of seven or eight species that preferred sites with deep water tables and less shade, and another set of eight to ten that preferred the opposite. The remaining nine to 11 species showed no clear pattern. These results confirmed the field impressions of the types of habitats in which these various species were abundant. Species such as *Pogonomyrmex badius, Pheidole adrianoi, Nylanderia arenivaga, T. septentrionalis, Camponotus socius*, and *Dorymyrmex bossutus* are conspicuous denizens of the high, dry, open habitats of the Florida sandhills, and are thus most abundant in the upper reaches of the flatwoods gradients. Their reduced occurrence in the very highest plots was probably the result of the greater canopy shading in those plots. Their preferred deeper water table is associated with the unpreferred greater shade. The relatively scarce *Temnothorax texanus* and the subterranean *Solenopsis nickersoni* also showed a preference for high, open sites. At the other extreme lay *N. faisonensis*, which occurred only in the lowest, shadiest sites, and was entirely absent from middle and upper sites. Almost as dramatic was the preference of *T. pergandei, S. carolinensis*, and *Crematogaster minutissima* for low, shady sites. Other species of these low, shady plots included *Formica archboldi* and *Cyphomyrmex rimosus*. For most of these species, it appears that preferred depth was associated with a level of openness that was not preferred. Interestingly, *F. pallidefulva* and *F. dolosa* were not significantly patterned by depth alone (Kruskal-Wallis test, Appendix 3), but when canopy shading and depth to water were separated as factors, both species were shown to prefer more shade in medium depth plots. In other words, the effects of depth and canopy were opposed.

**Figure 5.**
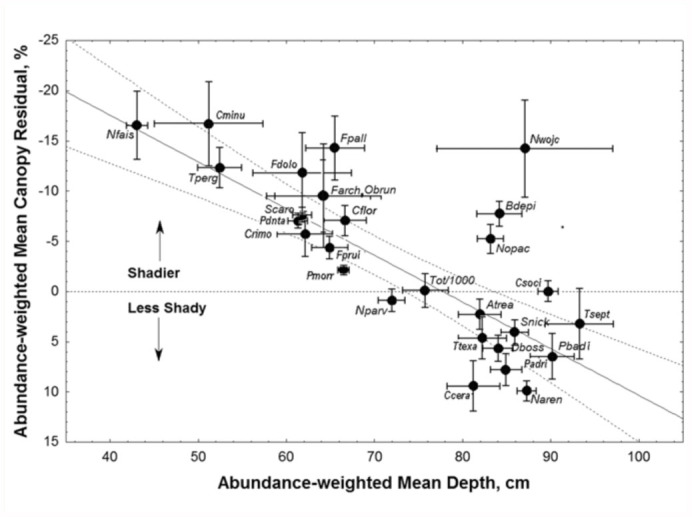
Species that were more frequent over shallow water tables also preferred shadier than average location for that depth. Species that were more frequent over deep water tables also preferred more open, sunny locations. See text for explanation of the variables. The regression was highly significant, and suggested that desiccation tolerance may be an attribute driving ant distribution. High quality figures are available online.

**Figure 6.**
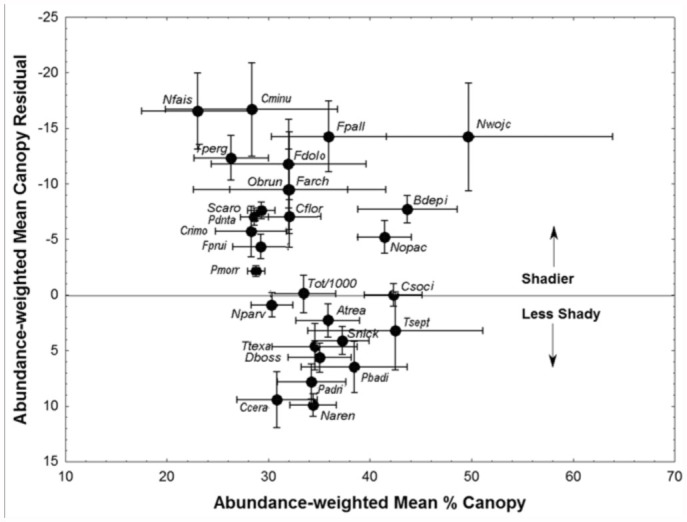
The depth adjusted, abundance-weighted percent canopy cover in relation to the actual abundance-weighted canopy cover. Species above the 0 line (i.e., negative residuals) are found in shadier than average areas no matter what the average shade is. Those below the line are found in sunnier locations relative to the average. The larger 95% confidence intervals for percent canopy. High quality figures are available online.

The patterns were reduced to a single metric by computing the weighted means for each plot and species. Because canopy cover is dependent on depth to water, canopy is, to a large degree, redundant with depth. However, by using the residuals from a regression of percent canopy cover against depth to water, the effect of shade on preference independent of the effect of depth to water was estimated. Weighted means were then calculated by entering each residual or depth value into the calculation once for every individual occurring in that plot, that is, by multiplying the plot depth and canopy values by the species' abundance. These abundanceweighted means (with 95% confidence intervals) are shown in Figures 5 and 6. In general, Figure 5 confirms the results of the ANOVAs, Mixed Procedures, and Figure 4, with species preferring low, shady plots (Talquin fine sand, see below) to the left, and high, open plots (Foxworth sand) to the right. The ants' preferred depth is associated with shadiness that is not preferred; it is open where depth is shallow, and shady where depth is deep. Species with a significantly negative mean residual canopy prefer more shade at the shallower depths at which they are abundant, whereas those with positive residuals are abundant at the greater depths with less shade, even though canopy cover increases with depth. This is probably why the plots with the greatest depth usually did not have the highest abundance of species preferring deep water tables. It may also be the reason why the middle zone in Figure 3 appears more densely populated than the extremes. The central region contains species that either preferred the middle ground or showed no significant preference.

The strong dependence of abundanceweighted residual canopy cover on depth to water suggested that temperature/moisture conditions are a basis for the distribution of many ant species. To the left in Figure 5 are species preferring the moist conditions created by a shallow water table and shading, while to the right are species that prefer the drier conditions created by a deep water table in well-drained soils along with open, sunny conditions. The former group of species seeks shade to increase moist conditions, whereas the latter seeks openness to increase dry conditions.

The weighted residual means were not significantly related to the weighted percent canopy means (Figure 6). However, across a wide range of canopy shade, shade-loving species clustered at negative residual values, whereas sun-loving species clustered at positive residuals. This indicates that no matter what the actual canopy cover, the former seek even shadier conditions and the latter more open.

**Figure 7.**
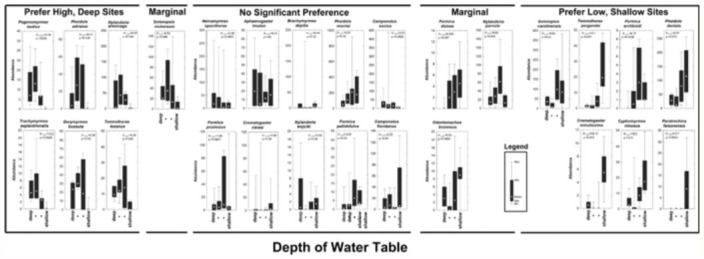
Median abundance, 75^th^ and 25^th^ percentiles in relation to the categories of depth to water. Species on the left occurred most abundantly over deeper water tables, those in the middle were more or less indifferent and those on the right occurred more often over shallow water tables. High quality figures are available online.

Figure 7 presents the median and percentile distribution of raw abundance in relationship only to depth to water, and is the graphical representation of the first table in Appendix 3. Many of the same distributions seen in Figures 4–6 are also visible in this figure, and once again, species fell along a continuum from those found most abundantly over deep water tables at the left to those over shallow water at the right, with species with no obvious preference between.

**Figure 8.**
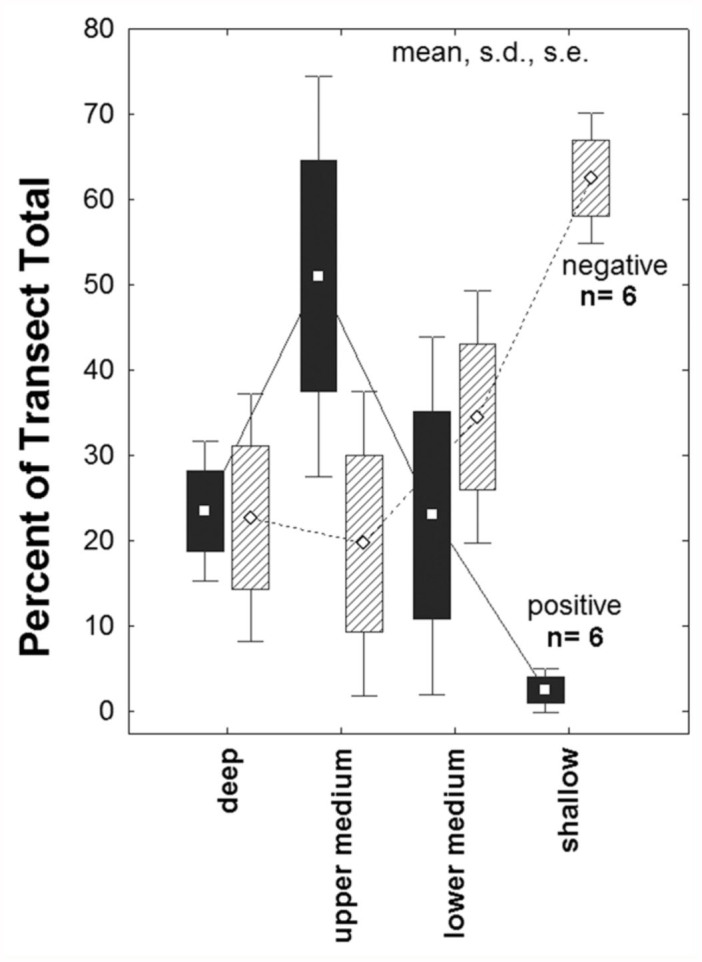
Species that were negatively correlated to factor 1 were relatively more abundant over shallow water tables, whereas positively correlated species were better represented over deeper water tables. For the included species, see Appendix 4. High quality figures are available online.

Because several species were distributed similarly, it was apparent that they must also be correlated to one another, and that it should be possible to reduce the number of variables through a factor analysis. This method extracts “factors” through a principal components procedure, and determines how strongly the values for each species are correlated to the extracted factors (Khattree and Naik 2000). For this analysis, the abundances were converted to the percent of the transect total of each species found at each of the four depths to water. The correlations for a two-factor analysis are shown in Appendix 4, and those species with correlations greater than 0.7 or -0.7 are shown in bold. Most species that showed a significant correlation in the factor analysis also showed non-random distribution patterns in the preceding ANOVAs and Mixed Procedure. Species that were significantly positively related to factor 1 were relatively more abundant over deeper water tables, whereas negatively correlated species were more abundant over shallow water tables (Figure 8). The avoidance of high canopy cover is once again visible for the species that prefer deep, open sites (i.e. correlate positively to factor 1).

### Vegetation

**Figure 9.**
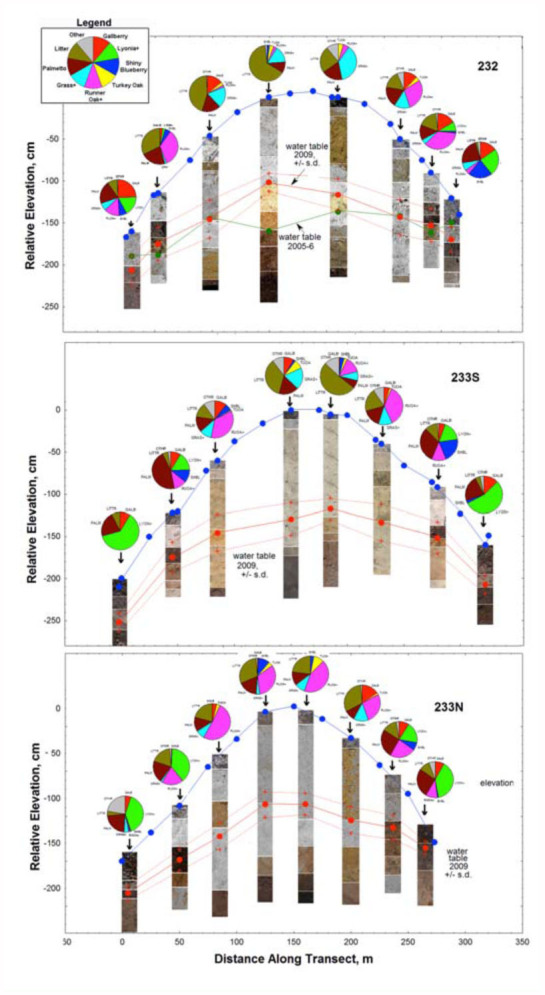
Soil profiles and pie diagrams of ground cover vegetation composition for the three transects. Soil from boring the wells was packed on a board, labeled with depth range, and photographed. Profiles were created from these digital images. The upper curve is the surface elevation along the transect, and the lower curves show the mean water table in 2009, plus and minus the standard deviation generated by water table fluctuation. The pie diagram at each test well location shows the composition of the ground cover vegetation within the sampled plots. In some cases, less common species were combined with strongly associated more common species. Such categories are followed by a “+”. Runner oak+ = runner oak combined with a much less common myrtle oak; Lyonia+ = fetterbush, rusty staggerbush, pepperbush, occasional titi, wax myrtle; gras+ = mostly wiregrass with occasional beardgrass; Other = bracken, huckleberry, St. John's wort. High quality figures are available online.

The depth to water also correlated generally with the vegetation (Figure 9, pie diagrams), although the composition at any particular site often consisted of different combinations of a set of plant species typical of that depth to water. Figure 9 shows that plots over shallow water table were dominated by *Lyonia* spp., gallberry, and palmetto in various proportions, and sometimes also harbored sweet pepperbush or wax myrtle. As the depth to the water table increased, this gave way to mixtures of runner oak, wiregrass, gallberry, and palmetto, as well as more litter-covered unvegetated area. Areas with the deepest water table sustained a mid-story of turkey oak, and a ground cover of wiregrass, shiny blueberry, litter, and runner oak. Turkey oak groves were found only where the water table averaged more than 1.0 m deep, and were much shadier than any of the other plots (see canopy images in Figure 1). Average canopy density increased from about 21% at the lowest elevations to 48% at the highest, a highly significant increase (ANOVA: F_3,20_ = 6.80; *p* < 0.005), but this pattern is not as clear in transect 233 N. Because turkey oaks are trees rather than ground cover plants, they did not appear at high frequency in the ground vegetation survey, thus their influence on conditions at ground level was underestimated by the vegetation survey.

**Figure 10.**
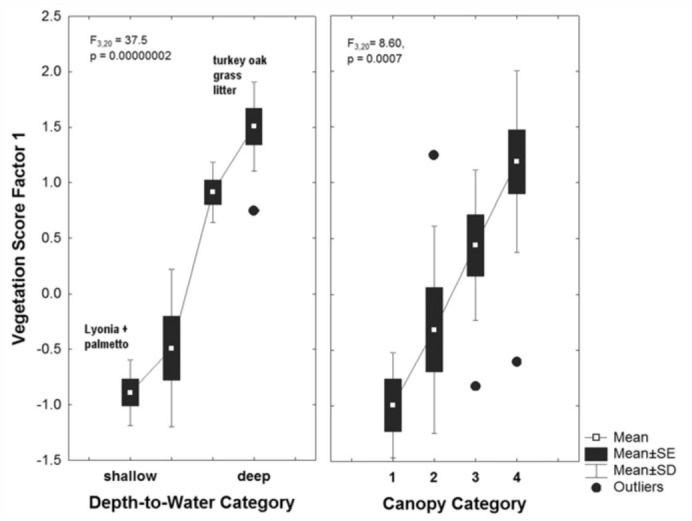
Factor 1 from a factor analysis of vegetation composition was strongly and positively related to both depth to water and percent canopy cover. Species over deep water and/or associated with deep shade were turkey oak, wiregrass, and leaf litter, whereas those over shallow water and open canopy were lyonia and similar species along with palmetto. High quality figures are available online.

A factor analysis of the most abundant ground cover species extracted three significant factors that together accounted for about 73% of the variance, and the first factor alone, 43%. The three factors together revealed 4 clusters of species: (1) turkey oak, grasses and litter; (2) runner oak; (3) gallberry, shiny blueberry, palmetto; (4) palmetto, lyonia. Palmetto was transitional between the last two groups, occurring abundantly in both. Species occurring over shallow water tables (lyonia, palmetto, etc.) had negative correlations to factor 1 that exceeded -0.7, and those over deep water tables (turkey oak, wiregrass, litter) correlated positively and were greater than 0.7. Thus, factor 1 scores increased strongly as water tables went from shallow to deep, and as sites became shadier (Figure 10), suggesting that the extracted groupings were reasonably consistent.

### Soils

Most soils of the Florida coastal plains were formed from sandy deposits on a marine platform, and are classified as Spodosols, or Entisols if they lack distinct horizons to > 2 m depth. These soils consist of unconsolidated sand that, because of prior weathering, has little potential for forming distinct horizons. Most have only a thin upper horizon, or epipedon. Spodosols have a deeper deposition layer, the spodic horizon, presumably formed when organic matter is precipitated through combination with aluminum and iron. The highest elevations of the transects in this study consisted of the moderately well-drained Foxworth sand, trending downward to the poorly-drained Talquin fine sand, and becoming the very poorly-drained Donovan mucky peat in the wetlands (USDA Web Soil Survey, http://websoilsurvey.nrcs.usda.gov/app/HomePage.htm).

The plots of soil profiles from the 24 wells were remarkably complex in their color variation (Figure 9). In all plots, the top 10–20 cm, the epipedon, was always grey, probably from fine charcoal deposited by frequent ground fires. This same layer contains about 85% of the total macronutrients in these nutrient-poor soils (Tschinkel, unpublished data). In general, as Talquin fine sand graded into the Donovan mucky peat of the wetlands, soils became darker and more organic, more solidified by deposited material, and lacking in light-colored layers. Soils from the higher elevations had large stretches of yellow or brownish to white sand underlain at greater depth by relatively solidified dark zones (spodic horizons) (Figure 9). Whereas these dark zones occurred in almost all cores (some may not have been deep enough), the depth of their occurrence seemed to show no clear pattern. That is, they did not form a smooth profile like the water table or surface elevation. In some cases, the hardened brown layer was underlain by pure white sand, sometimes below the water table. It seems likely that the location of this deposition layer was related to the water table, but what that relationship might be is currently not clear.

## Discussion

Moisture availability, governed largely by depth to water table, significantly impacts the standing biomass and species richness of plants in longleaf pine-wiregrass ecosystems because water is as limiting or more limiting than nutrient availability (Kirkman et al. 2001, Ford et al. 2008). Specifically, wet-mesic sites support greater floristic diversity and aboveground biomass than drier, upland sites (Kirkman et al. 2001, Ford et al. 2008). Ant abundance, however, appears to be highest in the sites near to the water table (lowermedium), but not the shallowest sites (Figures 3, 4). This suggests that ground-dwelling ant productivity is linked to total ecosystem productivity in these sites, but most species are limited by a water table near to the surface. Thus, depth to water table is one of the primary factors responsible for distribution patterns in both ants and plants, with the exception that ants are strongly constrained by the wettest sites (where plants are not). Obviously, water infiltration from below would constrain dwelling and rearing offspring in a subterranean nest. Beyond this simple generality, a variety of species-specific patterns emerged.

Well over half of the 27 more abundant ant species were significantly more abundant in some parts of the transects than in others. Several were entirely absent from either the highest or lowest locations. The transects represented gradients of depth to groundwater, vegetation, shading, elevation, soil differences, litter and other correlated features. The patterns of ant distributions thus correlate with a number of features, most of which correlate with each other. How can the causal factor(s) from this cafeteria of possibilities be teased out? To which feature(s) of the habitat are the ants responding, either by choices made by colonyfounding queens, migration, or differential survival of colonies? It seems doubtful that the species composition of the vegetation itself is critical. Gallberry vs. staggerbush may not matter. More likely, the vegetation creates a range of microhabitats of temperature, moisture, and food resources (such as insect prey) that lead to the patterned abundance of the ant species. Moreover, both the ants and the vegetation are likely responding directly to the depth to the water table. The vegetation gradient represents plants that range from obviously wetland or wetland boundary plants, to those that tolerate quite xeric conditions or are intolerant of moist conditions (i.e., *Opuntia* sp.).

At least some ants, if not responding directly to the plant cover, are probably responding directly to the depth to groundwater because the nests they normally excavate would be truncated by the water table. Some sandhill ant species, such as *S. geminata, D. bureni*, and *Prenolepis imparis*, excavate nests that are 4 m or more deep (Tschinkel 1987; Tschinkel 2003; unpublished data). These species are entirely absent from the flatwoods where the water table lurks never more than 2 m below ground. The Florida harvester ant, *Pogonomyrmex badius*, although constructing 2-3 m deep nests in the sandhills (Tschinkel 2004a) where groundwater is at > 4 m depth, seems more flexible, but is nevertheless limited to only the highest parts of the flatwoods landscape. This is also the case for *D. bossutus*. Of the other species preferring a deep water table, *T. septentrionalis* and *C. socius* excavate nests that are rarely more than 1.5 m deep (Tschinkel 2004b; Tschinkel 2005), so their preference for drier sites must have other reasons. The complete nest architectures of *P. adrianoi, T. texana, N. arenivaga* and *S. carolinensis* are currently not known.

Of the species that are more abundant over moderate to shallow water tables, only the architecture of *F. dolosa, F. archboldi* (Tschinkel, unpublished data), *F. pallidefulva* (Mikheyev and Tschinkel 2004), and *O. brunneus* (Cerquera and Tschinkel 2009) are known. All are less than 1.5 m deep. This is also true for several of the species showing no preference, including *P. morrisi* (Tschinkel 2003), *Aphaenogaster treatae* (Tschinkel 2011), and *C. floridanus* (Tschinkel, unpublished). It thus seems likely that depth to ground water is a possible controlling factor only for a few species that make very deep nests. However, location along the flatwood gradients also clearly affects soil moisture, which in turn may limit or create preferences for nest location. Soils near the tops of the gradients are more xeric (witness the frequent presence of prickly pear cactus), and are often well-drained, drying much faster after rains. In contrast, soils near the wetland margins often remain very wet for long periods. Some ants, such as *N. faisonensis, T. pergandei*, and *C. rimosus* seem to favor these wetter locations strongly. The great variability of desiccation resistance among ants (Hood and Tschinkel 1990) probably plays a role in these patterns of distribution, both when the ants are underground in their nests, and when foraging on the surface. The effect of soil moisture on brood, larvae in particular, may be particularly important. It is perhaps noteworthy that *Monomorium viride* thrives in hot dry sandhills and flatwood sites in spite of making nests that are never deeper than ∼40 cm (Tschinkel, unpublished data), emphasizing the complex relationships among nests, nest sites, and desiccation resistance. Spiesman and Cummings (2008) described the structuring of ant communities in Florida sandhills in relationship to local, regional, and landscape variation. Their results overlap ours at the local scale, and show generally similar patterns.

It stands to reason that unusual changes in the water table over several months should result in changes in the ant distribution. Indeed, populations of *T. septentrionalis* expanded during a record North Florida drought, and contracted again when that drought ended (Seal and Tschinkel 2010). The data on the response of the water table to rainfall (Figure 1, top panel) suggests that this change was associated with a rise in the water table, likely eliminating colonies closest to the wetland margins. The other ant species that were affected is unknown at this time.

With the exception of the dark spodic horizon and the surface layer, the color variation in the soil cores was not clearly associated with differences in soil structure. When dry, the surface layer was loose, and prone to easy disturbance. The spodic horizon ranged from somewhat firmer than the sand above, to hard enough to require a shovel for penetration. When *P. morrisi* dig through this spodic layer, the architectural features of their nests become compressed (Tschinkel, unpublished data). Chambers become smaller and closer together, but upon penetrating below this spodic layer, they resume the spacing and size present above the spodic layer. The effects of this layer on the architecture of other species are unknown.

The consistent, reduced abundance under the turkey oak canopy of several deepgroundwater species suggests that these species are adapted to and thrive in open, hot, and dry conditions. Conversely, a different set of species shuns the high/dry areas and thrives in the low/wet areas.

The clearly patterned distribution of ant species in relation to depth to water raises the question of how these patterns arise. Can foundresses discriminate among the rather subtle (to humans) differences in the soil, vegetation, shading, and elevation along these gradients? They would need to make this discrimination while still in flight, for once settled and wingless, they could not travel far afoot. Or do queens settle indiscriminately so that the pattern is created by differential survival along the flatwoods gradient? Possibly, mating and dispersal are very local, so that queen settlement declines rapidly as distance from the optimal habitat increases, assuring that colonization success drops off rapidly, preserving the observed abundance patterns. Competition tempered by habitat may also play a role, with species better able to compete in some parts of the gradient than others. Another possibility is that particular plant species may provide more food for particular ant species, either in the form of insect prey or exudations of Homoptera. At this time, it is not possible to choose among these (or other) alternatives. What remains is a pattern consisting of multiple strong correlations. The causes of the correlations are not known, but are amenable to experimental and observational analysis.

## Figures and Tables

**Appendix 1.**
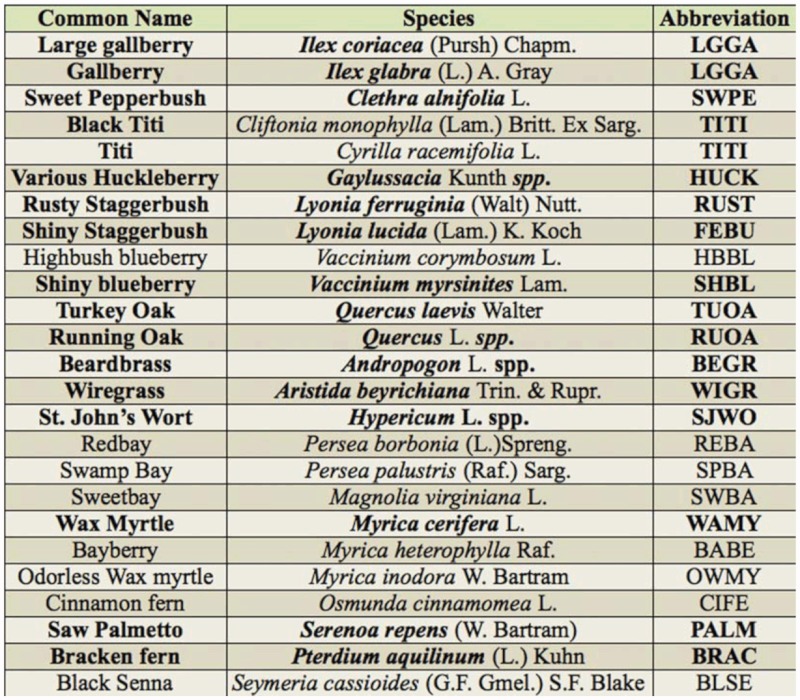
Common groundcover species of the piney flatwoods of the Apalachicola National Forest. Species found on the transects are shown in bold.

**Appendix 2.**
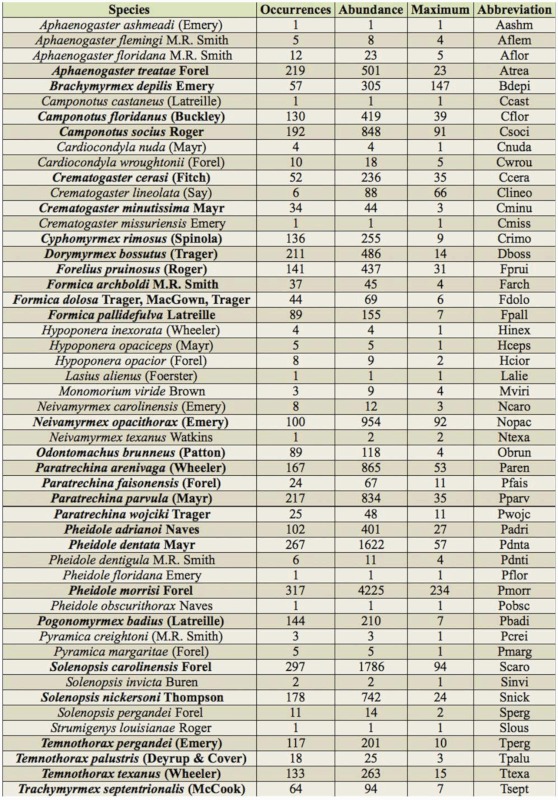
The species captured in pitfalls, their frequencies of occurrence, their total abundance summed over all traps, and the abbreviations used in figure labels. The maximum captured in a single trap is also shown. Species are arranged in alphabetical order. Species abundant enough to be used in the analysis are shown in bold.

**Appendix 3a.**
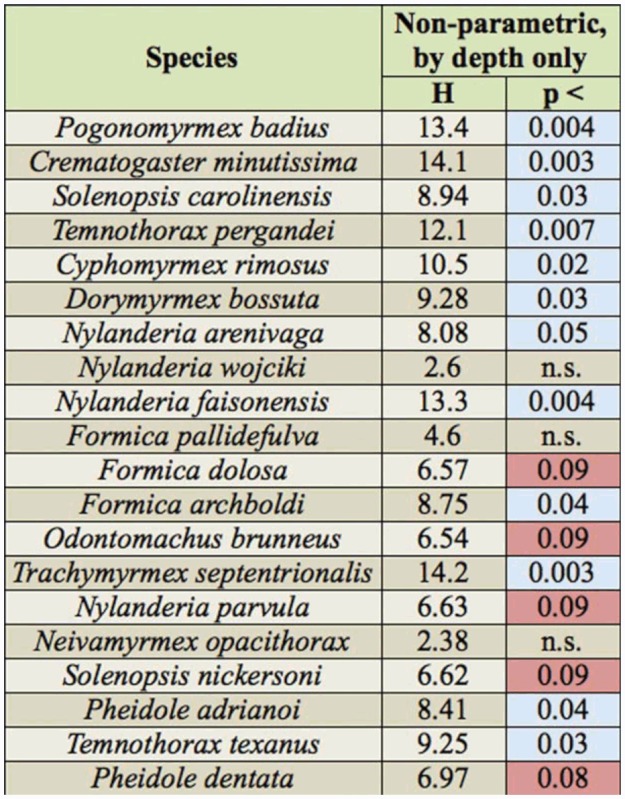
Non-parametric tests of species distribution in relation to depth to water only.

**Appendix 3b.**
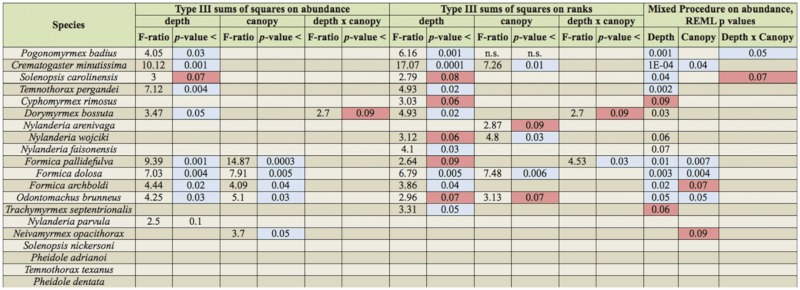
ANOVA results based on Type III sums of squares on ranks and abundance. For the Mixed Procedure, abundances were transformed to square root + 0.5 to meet the model assumptions. REML = residual maximum likelihood. Blue cells *p* < =0.05; red cells, *p* > =0.05 and < 0.10.

**Appendix 4.**
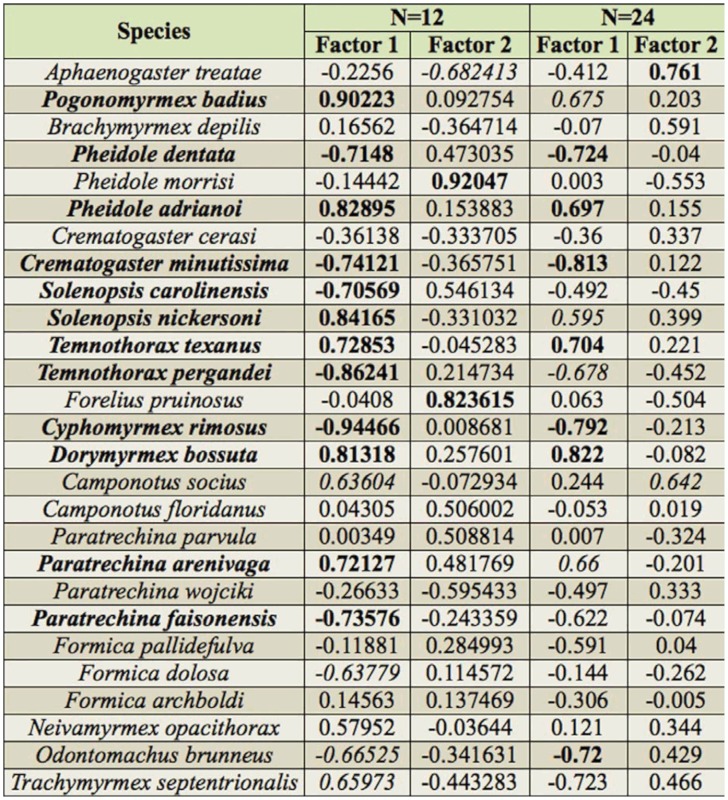
Unrotated factor analysis by principal components extraction: results for percent of the transect total of each species found at each depth to water category. Species correlated to factor 1 at > 0.70 are shown in bold, as are all correlations > -0.70. Correlations between 0.6 and 0.7 are shown in italics. The two plots within each transect that were at the same depth were combined for this analysis. Proportion of the total explained by factor 1 = 0.37, and by factor 2 = 0.18. Explained variance by factor 1 = 10.0 and by factor 2 = 4.74.
